# The role of small extracellular vesicle non-coding RNAs in kidney diseases

**DOI:** 10.3389/fgene.2022.1013637

**Published:** 2022-10-11

**Authors:** Chuxuan Luo, Haojie Liu, Lina Shao, Jiyu Tang, Qiang He, Juan Jin

**Affiliations:** ^1^ Urology & Nephrology Center, Department of Nephrology, Zhejiang Provincial People’s Hospital (Affiliated People’s Hospital, Hangzhou Medical College), Hangzhou, China; ^2^ Division of Health Sciences, Hangzhou Normal University, Hangzhou, China; ^3^ The 2nd Clinical Medical College, Zhejiang Chinese Medical University, Hangzhou, China; ^4^ Department of Nephrology, The First Affiliated Hospital of Zhejiang Chinese Medical University (Zhejiang Provincial Hospital of Traditional Chinese Medicine), Hangzhou, China

**Keywords:** biomarkers, diagnosis, kidney disease, non-coding RNAs, prognosis, small extracellular vesicles, therapeutics

## Abstract

Kidney diseases have become an increasingly common public health concern worldwide. The discovery of specific biomarkers is of substantial clinical significance in kidney disease diagnosis, therapy and prognosis. The small extracellular vesicle (sEV) can be secreted by several cell types, like renal tubular epithelial cells, podocytes, collecting duct cells and leap cells, and functions as a communication medium between cells by delivering signaling molecules, including proteins, lipids and nucleic acids. There has been growing evidence that kidney diseases are associated with aberrant expression of sEV-derived non-coding RNAs (sEV-ncRNAs). As a result, sEV-ncRNAs may provide valuable information about kidney diseases. In this paper, a systematic review is presented of what has been done in recent years regarding sEV-ncRNAs in kidney disease diagnosis, treatment and prognosis.

## 1 Introduction

Kidney diseases are a category of complicated disorders with diverse and uncertain etiologies. The diagnosis of kidney disease is commonly delayed, and patients often miss the best opportunity for treatment owing to its insidious onset and lack of a specific clinical manifestation in early stage. Patients with chronic kidney disease (CKD) are inextricably faced with dialysis and kidney transplant surgery as they progress to end-stage renal disease (ESRD). It has been estimated that there were approximately 4.9-7 million patients with ESRD requiring renal replacement therapy worldwide (Lv and Zhang, 2019). The reported annual treatment costs for dialysis patients accounted for 10% of total healthcare expenditure in China, which imposed significant financial burden on individuals, families, healthcare systems and society (Zhang and Wang, 2009).

As of now, percutaneous renal biopsy (PRB) is the only method used to definitively diagnose kidney diseases. Bleeding risks caused by PRB range in severity from transient gross hematuria to fatal (Bakdash et al., 2019). Due to these reasons, this examination is not performed in most cases. In addition to blood analyses (serum creatinine, blood urea nitrogen and creatinine clearance), urinalyses (urine density, proteinuria, hematuria, cylindruria and 24-h urine protein) are the currently widely used diagnostic markers in the clinical practice. Nevertheless, further examination is still needed to help clarify the pathological diagnoses of kidney diseases. As such, it is of paramount importance to develop novel non-invasive diagnostic methods for discriminating pathological types.

The exosome is a nano-sized extracellular vesicle of spherical shape, which was first discovered during the maturation of sheep reticulocytes in 1983 (Pan and Johnstone, 1983) and named by Johnstone et al. (1987) in 1987. A mature exosome forms by inward budding of endosomal membrane and then is released into the extracellular environment along with the plasma membrane (Amini et al., 2021). Recently, it has been revealed that exosomes with high purity were difficult to isolate. Accordingly, the International Society for Extracellular Vesicle recommended referring to vesicle with a diameter less than 200 nm as small extracellular vesicle (sEV) (Théry et al., 2018). Nearly all kidney inherent cells release sEVs, including renal tubular epithelial cells, podocytes, collecting duct cells and leap cells (Miranda et al., 2010). Moreover, they can be found in multiple body fluids, such as blood, urine, saliva, cerebrospinal fluid and milk (van Niel et al., 2018). There have been various molecular components found in sEVs, such as proteins, lipids and nucleic acids, which are protected from external environment by the lipid bilayer membrane structures of sEVs (Kim et al., 2017; Jeppesen et al., 2019). These molecules vary greatly with the pathophysiological conditions of parent cells (Pegtel and Gould, 2019). Accumulating evidence has revealed that sEVs were rich in non-coding RNAs (ncRNAs), like microRNAs (miRNAs), long non-coding RNAs (lncRNAs) and circular RNAs (circRNAs), which are involved in various disease pathophysiology (Liu et al., 2019; Ren and Wang, 2021).

**FIGURE 1:**
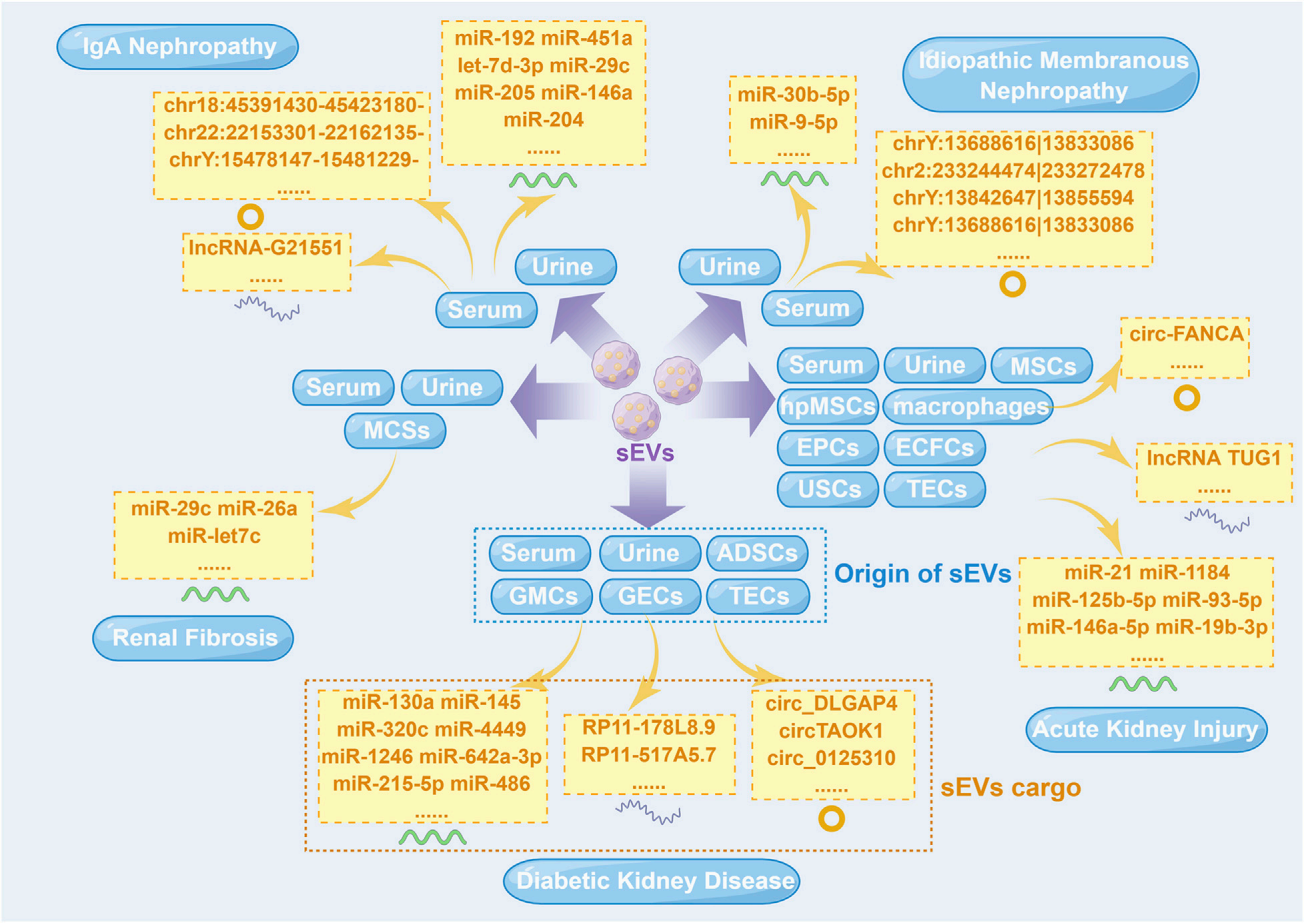
Small extracellular vesicle non-coding RNAs isolated from different cells in five major kidney diseases. ADSCs, adipose-derived stem cells; ECFCs, endothelial colony-forming cells; EPCs, endothelial progenitor cells; GECs, glomerular endothelial cells; GMCs, glomerular mesangial cells; hpMSCs, human placental mesenchymal stem cells; lncRNA, long non-coding RNA; miR, microRNA; MSCs, mesenchymal stem cells; sEVs, small extracellular vesicles; TECs, tubular epithelial cells; USCs, urine-derived stem cells. This figure was created by Figdraw (www.figdraw.com) (ID: YWWOI4c8de).

In the last few years, a number of previous studies have demonstrated a close relationship between sEV-derived ncRNAs (sEV-ncRNAs) and kidney diseases, providing innovative insights for their diagnosis and treatment (Ichii and Horino, 2018). Different sEV-ncRNAs have different mechanisms in kidney diseases (Figure 1). This review is primarily concerned with providing an overview of sEV-ncRNAs and their potential use in five major kidney disease, including diabetic kidney disease (DKD), acute kidney injury (AKI), IgA nephropathy (IgAN), idiopathic membranous nephropathy (IMN) and renal fibrosis.

## 2 Roles of sEV-ncRNAs in DKD

There are several microvascular complications associated with diabetes, but the most prevalent and serious is DKD, which has become a major cause of ESRD (Alicic et al., 2017). Although PRB remains the gold standard diagnostic criteria for DKD at the present time, there is a certain amount of controversy regarding its indications in clinical practice. Accordingly, the diagnosis of DKD is made on clinical presentation in the majority of the cases. But the clinical diagnosis of DKD is challenging in clinical use and a misdiagnosis rate has been reported to be as high as 49.2% in a recent meta-analysis (Fiorentino et al., 2017). For this reason, the need for early detection and intervention of DKD becomes an imperative issue. Increasing number of studies revealed that sEV-ncRNAs contributed to the progression of DKD, even serving as biomarkers for diagnostics and therapeutic purposes (Table 1).

**TABLE 1:** The role of small extracellular vesicle non-coding RNAs in diabetic kidney disease.

Origin of sEVs	sEVs cargo	Pathway	ncRNA expression	Mechanism	References
urine samples GMCs	miR-145	N/A	high	represent a novel candidate diagnostic biomarker for diabetic kidney disease	Barutta et al. (2013)
urine samples	miR-320c	TGF-β signaling pathway	high	represent a novel candidate diagnostic biomarker for diabetic kidney disease	Delić et al. (2016)
ADSCs	miR-215-5p	ZEB2	high	protect against high glucose-induced metastasis	Jin et al. (2020)
ADSCs	miR-486	Smad1/mTOR signaling pathway	high	lead to the increase of autophagy and the reduction of podocyte apoptosis	Jin et al. (2019)
serum samples	miR-1246	MAPK signaling pathway	high	represent a novel candidate diagnostic biomarker for diabetic kidney disease	Kim et al. (2019)
	miR-642a-3p				
	let-7c-5p				
	miR-1255b-5p				
	let-7i-3p				
	miR-5010-5p				
	miR-150-3p				
	miR-4449				
urine samples	miR-21-5p	N/A	high		
	miR-30b-5p		low	represent a novel candidate diagnostic biomarker for diabetic kidney disease	Zang et al. (2019)
TECs	miR-6724-5p	N/A	high	represent a novel candidate diagnostic biomarker for diabetic kidney disease	Zhou et al. (2021)
	miR-6716-3p				
	miR-2355-3p				
	miR-135b-3p				
	miR-3180				
	miR-5008-3p		low		
	miR-6785-5p				
	miR-3654				
	miR-335-3p				
	RP11-178L8.9		high		
	CTD-2530H12.2				
	RP11-503N18.4				
	RP11-20B24.7				
	RP11-256I23.1				
	RP11-517A5.7		low		
	RN7SL870P				
	CTD-2298J14.2				
	ANKRD10-IT1				
	AP000442.1		low		
	circRNA_164				
	circRNA_225				
	circRNA_57				
GMCs	circ_DLGAP4	miR-143/ERBB3/NF-κB/MMP2 axis	high	promote proliferation and fibrosis of GMCs	Bai et al. (2020)
GECs	circTAOK1	miR-520h/Smad3 axis	high	promote proliferation, fibrosis, and EMT of GMCs	Li et al. (2022a)
GMCs	circ_0125,310	miR-422a/IGF1R/p38 axis	high	promote proliferation and fibrosis of GMCs	Zhu et al. (2022)

ADSCs, adipose-derived stem cells; circRNA, circular RNA; EMT, epithelial-mesenchymal transition; ERBB3, Erb-b2, receptor tyrosine kinase 3; GECs, glomerular endothelial cells; GMCs, glomerular mesangial cells; IGF1R, insulin-like growth factor 1 receptor; MAPK, mitogen-activated protein kinase; miR, microRNA; MMP2, matrix metalloproteinase 2; mTOR, mechanistic target of rapamycin; N/A, not available; ncRNA, non-coding RNA; NF-κB, nuclear factor-κB; sEVs, small extracellular vesicles; Smad1, SMAD, family member 1; Smad3, SMAD, family member 3; TECs, tubular epithelial cells; TGF-β, transforming growth factor-β; ZEB2, zinc finger E-box-binding homeobox 2.

### 2.1 Roles of sEV-miRNAs in DKD

The latest research suggested the diagnostic value of aberrant expression of sEV-derived miRNAs (sEV-miRNAs) in DKD. According to Barutta et al. (2013), the expression of urinary sEV-derived miR-130a as well as miR-145 was markedly elevated in type 1 diabetic mellitus (T1DM) patients with microalbuminuria than health donors and those without microalbuminuria. Researchers further verified their results by cell-based and animal experiments. miR-145 expression was more than nine-fold higher in diabetic mice’s glomerulus and two-fold higher in their urine sEVs as compared to healthy mice. Similarly, high glucose conditions increase the expression of miR-145 in glomerular mesangial cells (GMCs). These results suggested the diagnostic value of miR-145 applied in DKD early diagnosis. Another study from Delić et al. (2016) has revealed that fourteen upregulated urinary sEV-miRNAs (miR-320c, miR-6068, miR-1234-5p, miR-6133, miR-4270, miR-4739, miR-371b-5p, miR-638, miR-572, miR-1227-5p, miR-6126, miR-1915-5p, miR-4778-5p and miR-2861) and two downregulated urinary sEV-miRNAs (miR-30d-5p and miR-30e-5p) were significantly differentially expressed in DKD patients as compared to health donors and type 2 diabetes mellitus (T2DM) patients. Among these miRNAs, miR-320c, the most strongly upregulated miRNA in urine sEVs from DKD patients, may be involved in the development of DKD through transforming growth factor-β (TGF-β) signaling pathway. Considering the above study results, miR-320c appeared to represent an intriguing candidate marker for the diagnosis of DKD, which needed to be further investigated. As well, Kim et al. (2019) analyzed sEV-miRNAs derived from serum samples of healthy donors as well as diabetics with and without nephropathy. They observed a significant upregulation of seven sEV-miRNAs (miR-1246, miR-642a-3p, let-7c-5p, miR-1255b-5p, let-7i-3p, miR-5010-5p and miR-150-3p) in DKD patients compared to health donors and a dramatic upregulation of miR-4449 in DKD patients in comparison with patients who do not have DKD. It was determined that the above-mentioned miRNAs play a role in mitogen-activated protein kinase (MAPK) signaling, integrin function in angiogenesis, and activator protein 1 (AP-1) transcription factor regulation. The authors thus concluded that these miRNAs are expected to be targets for DKD diagnosis. Beyond these, Zang et al. (2019) found that urinary sEV-derived miR-21-5p was upregulated and miR-30b-5p was downregulated in DKD individuals compared to controls, pointing out possible functions of these miRNAs as diagnostic biomarkers for DKD.

Furthermore, miRNA-containing sEVs may be effective in the therapy of DKD. In our laboratory, sEVs from adipose-derived stem cells (ADSCs) were demonstrated to be a mediator of miR-215-5p uptake by podocytes, thus preventing glucose-induced metastasis, possibly by inhibiting of zinc finger E-box-binding homeobox 2 (ZEB2) transcription (Jin et al., 2020). Another study from our research group found that sEVs derived from ADSCs improved the DKD patients’ symptoms through inhibiting SMAD family member 1 (Smad1)/mechanistic target of rapamycin (mTOR) signaling pathway by enhancing miR-486 expression in podocytes (Jin et al., 2019). These finding illustrated that sEV derived from ADSCs was a potential therapeutic strategy for DKD.

### 2.2 Roles of sEV-lncRNAs in DKD

At present, the study toward the role of sEV-derived lncRNAs (sEV-lncRNAs) in early diagnosis of DKD has only just begun to make headway. According to Zhou et al. (2021), lncRNAs were expressed differently in sEVs from human renal tubular epithelial cells (TECs) that were treated or not with high glucose. There were a total of 169 lncRNAs differentially expressed in sEVs, of which 93 were upregulated and 76 were downregulated. Among these, the top five upregulated lncRNAs were RP11-178L8.9, CTD-2530H12.2, RP11-503N18.4, RP11-20B24.7 and RP11-256I23.1, while the top five downregulated lncRNAs were RP11-517A5.7, RN7SL870P, CTD-2298J14.2, ANKRD10-IT1 and AP000442.1, respectively. Afterward, investigators further performed Kyoto Encyclopedia of Genes and Genomes (KEGG) enrichment analyses to investigate potential functions of sEV-lncRNAs with differential expression. It was found that lncRNAs were involved in such pathways as T2DM. Preliminary results from this study demonstrated that the aforementioned sEV-lncRNAs might contribute to the progression of DKD, and may also be served as biomarkers in this disease.

### 2.3 Roles of sEV-circRNAs in DKD

**FIGURE 2:**
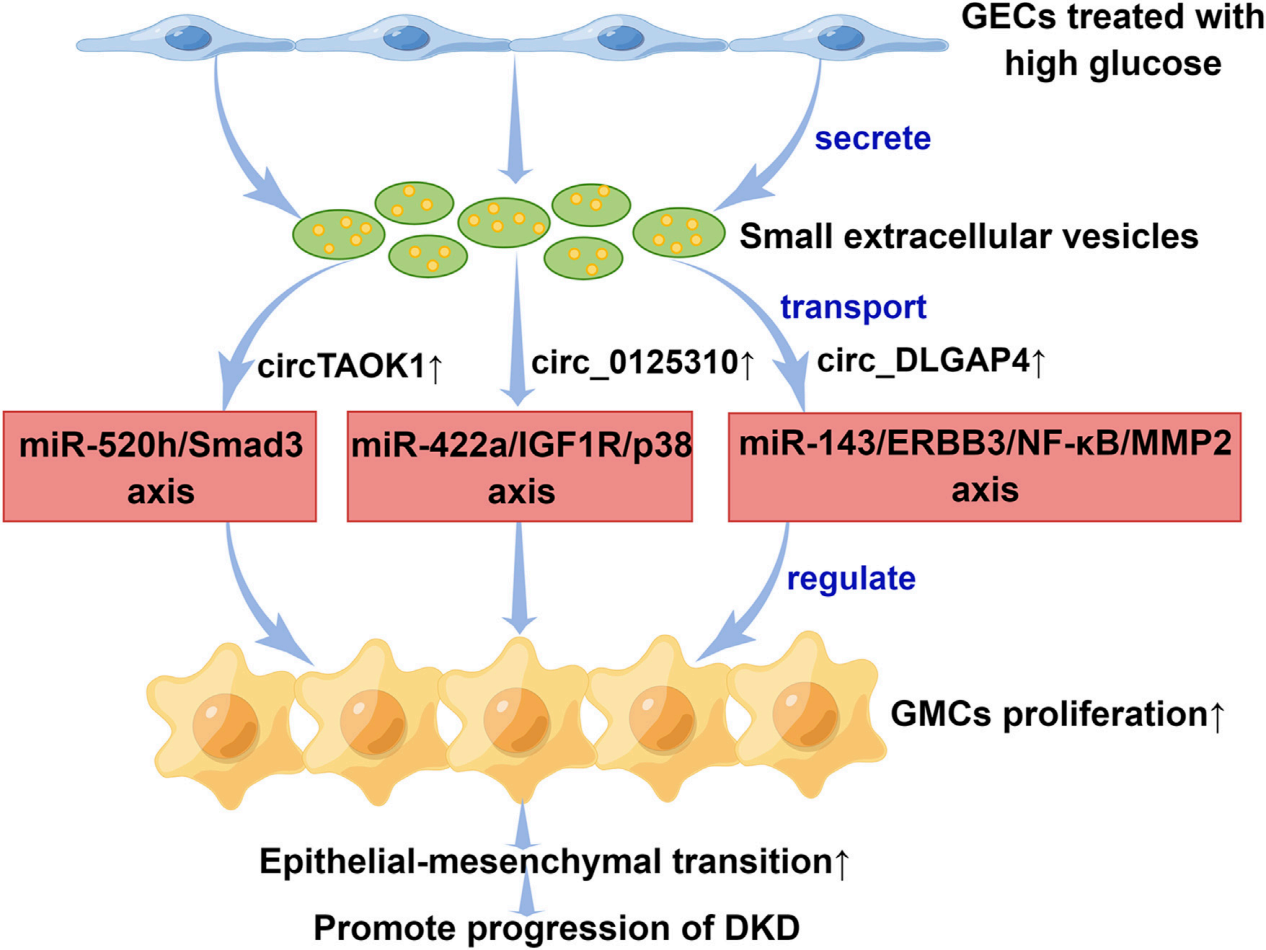
The mechanisms of small extracellular vesicle circular RNAs in the progression of diabetic kidney disease. Small extracellular vesicles secreted by GECs promoted epithelial interstitial transition process in diabetic kidney disease through transporting different circular RNAs. DKD, diabetic kidney disease; ERBB3, Erb-b2 receptor tyrosine kinase 3; GECs, glomerular endothelial cells; GMCs, glomerular mesangial cells; IGF1R, insulin-like growth factor 1 receptor; miR, microRNA; MMP2, matrix metalloproteinase 2; NF-κB, nuclear factor-κB; Smad3, SMAD family member 3. This figure was created by Figdraw (www.figdraw.com) (ID: PWOUSd94f7).

circRNAs are a special class of ncRNAs and one of the current research hotspots of scholars in various countries. There has been an increase in the number of studies that focus on sEV-derived circRNAs (sEV-circRNAs) in DKD pathophysiology over the past few years (Figure 2). Interestingly, the study of Li et al. (2022) indicated that when high glucose is applied to glomerular endothelial cells (GECs), the expression of circTAOK1 (also known as circ_0003928) is upregulated. Mechanically, circTAOK1 was identified to accelerate GMCs proliferation and epithelial interstitial transition (EMT) *via* targeting the miR-520h/SMAD family member 3 (Smad3) axis. In addition, high expression of circ_0125310 have been found in sEVs from high glucose-induced GMCs in a study by Zhu et al. (2022). They revealed that circ_0125310 could promote GMCs proliferation and fibrosis in DKD through sponging miR-422a and activating the insulin-like growth factor 1 receptor (IGF1R)/p38 pathway. Bai et al. (2020) demonstrated that circ_DLGAP4 was significantly upregulated in sEVs from high glucose-treated GMCs, which led to an increase in the progression of DKD by modulating miR-143/Erb-b2 receptor tyrosine kinase 3 (ERBB3)/nuclear factor-κB (NF-κB)/matrix metalloproteinase 2 (MMP2). Hopefully, these results will help scientists gain a better understanding of DKD pathogenesis.

## 3 Roles of sEV-ncRNAs in AKI

Clinically, AKI is the result of a brief duration of prerenal, renal, or postrenal injury, which is closely associated with poor prognosis of hospitalized patients (Liangos et al., 2006). It is currently diagnosed primarily by serum creatinine and urinary output, according to the Kidney Disease Improving Global Outcomes clinical practice guideline (Khwaja, 2012). However, several investigators have argued that the diagnosis of AKI made by measuring serum creatinine levels was problematic since serum creatinine levels tend to rise after renal function declines and do not indicate injury (Slocum et al., 2012). Consequently, the search for sensitive, reliable and early biomarkers represent urgent topics to be explored in the future. sEV-ncRNAs have been reported to participate in the development of AKI and could be served as specific biomarkers for AKI (Table 2).

**TABLE 2:** The role of small extracellular vesicle non-coding RNAs in acute kidney injury.

Origin of sEVs	sEVs cargo	Pathway	ncRNA expression	Mechanism	References
MSCs	miR-125b-5p	p53	high	promote tubular repair	Cao et al. (2021)
serum samples	miR-181a-5p and miR-23b-3p	N/A	high	represent a novel candidate diagnostic biomarker for acute kidney injury	Da-Silva et al. (2022)
serum samples	miR-500a-3p	MLKL	high	suppress cell injury and inflammation	Jiang et al. (2019)
USCs	miR-146a-5p	IRAK1	high	protect TECs from H/R injury	Li et al. (2020)
TECs	miR-19b-3p	NF-κB/SOCS-1	high	promote M1 macrophage activation	Lv et al. (2020)
ECFCs	miR-486-5p	PTEN/Akt signaling pathway	high	reduce ischemic kidney injury	Viñas et al. (2016)
TECs	miR-20a-5p	N/A	high	inhibit mitochondrial injury and apoptosis of TECs	Yu et al. (2020)
urine samples	miR-21	N/A	high	have good accuracy in the diagnosis of diabetic kidney disease	Yun et al. (2021)
MSCs	miR-1184	FOXO4, p27 Kip1 and CDK2	high	induce G1 phase arrest in TECs	Zhang et al. (2021a)
hPMSCs	miR-93-5p	N/A	high	protect from progression of I/R	Zhang et al. (2022a)
EPCs	miR-21-5p	RUNX1	high	improve renal function and renal tissue pathological damage, attenuate serum inflammatory response, as well as reduce apoptosis and oxidative stress response in renal tissues, and regulate endothelial glycocalyx damage marker proteins syndecan-1 and heparanase-1	Zhang et al. (2021b)
macrophages	miR-155	NF-κB/SOCS-1	high	mediate the communication between activated macrophages and injured tubules	Zhang et al. (2022b)
USCs	lncRNA TUG1	SRSF1	high	regulate ASCL4-mediated ferroptosis	Sun et al. (2022)
TECs	circ-FANCA	miR-93-5p/OXSR1 axis	high	alleviate LPS-induced HK2 cell injury	Li et al. (2021)

ASCL4, acyl-CoA, synthetase long-chain family member 4; CDK2, cyclin dependent kinase 2; ECFCs, endothelial colony-forming cells; EPCs, endothelial progenitor cells; FOXO4, forkhead box O4; hPMSCs, human placental MSCs; H/R, hypoxia-reoxygenation; I/R, ischemia-reperfusion; IRAK1, interleukin-1, receptor-associated kinase 1; lncRNA, long non-coding RNA; LPS, lipopolysaccharide; miR, microRNA; MLKL, mixed lineage kinase domain-like; MSCs, mesenchymal stem cells; N/A, not available; ncRNA, non-coding RNA; NF-κB, nuclear factor-κB; OXSR1, oxidative stress responsive 1; PTEN, phosphatase and tensin homolog; RUNX1, runt-related transcription factor 1; sEVs, small extracellular vesicle; SOCS-1, suppressor of cytokine signaling 1; SRSF1, serine/arginine splicing factor 1; TECs, tubular epithelial cells; USCs, urine-derived stem cells.

### 3.1 Roles of sEV-miRNAs in AKI

sEV-miRNA can be isolated from most body fluids and is one of the research hot spots in the field of AKI diagnosis, with a promising future in liquid biopsy. As one of the most studied miRNAs, urinary sEV-derived miR-21 levels were increasingly recognized as diagnostic predictors of AKI. The analysis of urinary sEV-miRNA expression in 25 scrub typhus patients with and without AKI was performed by Yun et al. (2021), and revealed significantly higher expression levels of miR-21 in the AKI patients compared to controls. In a receiver operating characteristic (ROC) curve analysis, urinary sEV-derived miR-21 was demonstrated to have good discriminative power for the diagnosis of scrub typhus-associated AKI, with an area under the curve (AUC) of 0.908, suggesting good diagnostic potential. Moreover, the expressions of serum sEV-derived miR-181a-5p and miR-23b-3p were found to be increased in lipopolysaccharide (LPS)-induced AKI mouse models (Da-Silva et al., 2022). It has been shown by bioinformatics analysis that both miRNAs target transcription factors that regulate genes expressing proinflammatory cytokines. These findings yield great promise for a future of precision medicine as sEV-miRNAs might be used clinically to identify patients with AKI early.

For now, numerous important advances regarding the therapeutic potential of sEV-miRNAs in AKI have been reported. As an example of this, Cao et al. (2021) confirmed that sEV-miR-125b-5p from mesenchymal stem cells (MSCs) could stimulate tubular repair by suppressing p53 in AKI. Similarly, our laboratory discovered that sEV-miR-1184 from MSCs can mitigate cisplatin-associated AKI (Zhang et al., 2021). Additionally, sEVs from three-dimensional (3D) cultures of human placental MSCs (hPMSCs) have been reported to be effective for the treatment of AKI (Zhang et al., 2022). A significant change in miR-93-5p was found in sEVs obtained from hPMSCs cultured in 3D. Hence, it is easily conceivable that sEVs from 3D culture of hPMSCs might exert therapeutic effects through miR-93-5p modulation, but its specific molecular mechanism remains unknown currently. Moreover, Zhang et al. (2021) discovered that miR-21-5p-containing sEVs derived from endothelial progenitor cells (EPCs) were able to alleviate sepsis-induced AKI by silencing runt-related transcription factor 1 (RUNX1). Urine-derived stem cells (USCs) have also been shown to perform a protective effect on ischemic/reperfusion injury (IRI)-induced AKI *via* sEV-derived miR-146a-5p targeting interleukin-1 receptor-associated kinase 1 (IRAK1) in a recent study (Li et al., 2020). Apart from these, miR-486-5p-enriched sEVs from endothelial colony-forming cells (ECFCs) (Viñas et al., 2016), miR-500a-3p-enriched sEVs from serum samples (Jiang et al., 2019), as well as miR-20a-5p-enriched sEVs from TECs (Yu et al., 2020) have all been considered the promising therapies for AKI.

In addition, several sEV-miRNA association studies have offered new insights into the pathogenesis of AKI. Lv et al. (2020) have originally found that sEV-miR-19b-3p derived from TECs facilitated M2 macrophage activation *via* targeting the NF-κB/suppressor of cytokine signaling 1 (SOCS-1) in LPS-induced AKI. Another study in China discovered that sEV-derived miR-155 promoted the progression of AKI by mediating communication between activated macrophages and damaged tubules. The results of all of these studies have contributed to our understanding of AKI’s pathophysiology (Zhang et al., 2022).

### 3.2 Roles of sEV-lncRNAs in AKI

Recently, there is preliminary evidence of a positive therapeutic effect of sEV-lncRNAs in patients with AKI. The latest discovery from Sun et al. (2022) has revealed that IRI-induced AKI was attenuated by lncRNA TUG1 derived from USCs that inhibited acyl-CoA synthetase long-chain family member 4 (ASCL4)-mediated ferroptosis through interaction with serine/arginine splicing factor 1 (SRSF1). Hence, lncRNA TUG1 might represent an ideal target for developing a AKI therapeutic.

### 3.3 Roles of sEV-circRNAs in AKI

sEV-circRNAs have been found associated with the pathogenesis of AKI. It is known that those suffering from septic shock are more likely to experience AKI in the intensive care unit. LPS-induced sepsis is the leading cause of AKI in critically ill patients (Netti et al., 2019). It was discovered that sepsis-induced AKI was characterized by a high expression of circ-FANCA, which was generated by precursor mRNA FANCA (Kölling et al., 2018). As yet, it remains unclear how circ-FANCA contributes to the pathogenesis of sepsis-induced AKI. In recent years, investigators found that sEV-derived circ-FANCA was able to directly interact with miR-93-5p and regulate oxidative stress responsive 1 (OXSR1) expression, thus modulating LPS-induced TECs injury (Li et al., 2021). The results provided a potential therapeutic target for septic AKI.

## 4 Roles of sEV-ncRNAs in IgAN

IgAN currently represents the most prevalent form of primary glomerulonephritis worldwide with IgA deposition within the glomerular mesangium (Lai et al., 2016). IgAN may occur in any age group but typically between age 16 and 35, which has been reported to be one of the leading causes of ESRD in young adults (Huang and Xu, 2021). Although histological confirmation is still necessary for the diagnosis of IgAN, this approach has a limited indication for clinical application till now. Regrettably, no specified laboratory markers for identifying IgAN are available up to now. In the following, we have summarized the latest advances in the emerging role of sEV-ncRNAs in kidney diseases (Table 3).

**TABLE 3:** The role of small extracellular vesicle non-coding RNAs in IgA nephropathy.

Origin of sEVs	sEVs cargo	Pathway	ncRNA expression	Mechanism	References
serum samples	miR-192	N/A	low	accelerate decline in renal function	Fan et al. (2019)
urine samples	miR-451a	N/A	high	regulation of mRNA stability, extracellular exosome and transferase activity	Li et al. (2022b)
urine samples	miR-29c	N/A	low		
	miR-205				Min et al. (2018)
	miR-146a		high		
urine samples	miR-204	N/A	low	N/A	Pawluczyk et al. (2021)
serum samples	lncRNA-G21551	N/A	low	N/A	Guo et al. (2020)
urine samples	chr18:45391430-45423180-	PI3K/Akt signaling pathway	high	regulate primary miRNA processing, the ability of angiotensin receptor binding, and stress fibre function	Luan et al. (2021)
	chr22:22153301-22162135-				
	chr1:243708812-243736350-				
	chr19:42740758-42744294-				
	chr17:11984673-12016677+				
	chr18:11862394-11876687+				
	chr17:8409636-8413,267-				
urine samples	chrY:15478147-15481229-	N/A	high	alter the expression of the coding gene UTY protein	Luan et al. (2022)

LncRNA, long non-coding RNA; mRNA, messenger RNA; miR/miRNA, microRNA; N/A, not available; ncRNA, non-coding RNA; PI3K, phosphoinositide-3-kinase-protein kinase B; sEVs, small extracellular vesicles.

### 4.1 Roles of sEV-miRNAs in IgAN

sEV-miRNAs have shown diagnostic potential in early diagnosis of IgAN in various studies. Min et al. (2018) found that the urinary sEVs of IgAN patients expressed significantly lower levels of miR-29c and miR-205 than did those of healthy controls, while miR-146a was significantly higher. In a study by Pawluczyk et al. (2021), the miR-204 expression was significantly decreased in urine sEVs from the patients with IgAN compared to healthy controls. Li et al. (2022) discovered that there existed significant differences in the expression of miR-451a and let-7d-3p in urine sEVs between IgAN patients and healthy individuals. To assess the diagnostic performance of these miRNAs, researchers further carried out ROC curve analysis and concluded that miR-451a and let-7d-3p can be served as biomarkers for early diagnosis of IgAN. These results revealed that specific sEV-miRNAs may be used as non-invasive biomarkers for the detection of IgAN. Despite these very promising results, validation in large sample cohorts is still pending.

sEV-miRNAs are related not only to the early diagnosis but also to the prognosis of IgAN. A study comprising 50 IgAN patients and 25 healthy control individuals indicated that the higher the expression level of serum miR-192 was, the less likely the patient is to have renal function decline within 2 years (Fan et al., 2019). This observation illustrated that miR-192 possessed great potential to become a prognostic biomarker in IgAN.

### 4.2 Roles of sEV-lncRNAs in IgAN

There have been relatively few studies on the potential role of sEV-lncRNAs in IgAN diagnosis. A recent study found that among IgAN patients, serum sEV-derived lncRNA-G21551, with the closest protein coding gene, was markedly downregulated, suggesting its promising diagnostic value (Guo et al., 2020).

### 4.3 Roles of sEV-circRNAs in IgAN

The study regarding sEV-circRNAs involvement in the diagnosis of IgAN is only just emerging from its infancy. Luan et al. (2021) have showed a total of seven urinary sEV-circRNAs (circRNAchr18:45391430-45423180-, circRNAchr22:22153301-22162135-, circRNAchr1:243708812-243736350-, circRNAchr19:42740758-42744294-, circRNAchr17:11984673-12016677+, circRNAchr18:11862394-11876687+ and circRNAchr17:8409636-8413,267-) were significantly upregulated in IgAN patients. Gene ontology (GO) analysis demonstrated that the above-mentioned sEV-circRNAs may be involved in primary miRNA processing, the ability of angiotensin receptor binding, and stress fiber function. According to KEGG analysis, these sEV-circRNAs are likely closely related to the phosphoinositide-3-kinase-protein kinase B (PI3K)/Akt signaling pathway. Another study from the same research group found that the expression of urinary sEV-circRNA chrY:15478147-15481229- derived from sex chromosomes was remarkably upregulated in male patients with IgAN (Luan et al., 2022). Thus, the authors concluded that these sEV-circRNAs holds promise as novel biomarkers in the early diagnosis of IgAN.

## 5 Roles of sEV-ncRNAs in IMN

Idiopathic membranous nephropathy (IMN) is one of the principal causes of primary nephrotic syndrome in adults, which is characterized by the accumulation of immune complexes between podocytes and the basement membranes of the glomerulus. Nowadays, the diagnosis of IMN is made based on pathologic confirmation. A specific biomarker for accurate diagnosis of IMN is still lacking. Herein, we have provided a brief summary of latest research on sEV-ncRNAs in IMN (Table 4).

**TABLE 4:** The role of small extracellular vesicle non-coding RNAs in idiopathic membranous nephropathy.

Origin of sEVs	sEVs cargo	Pathway	ncRNA expression	Mechanism	References
urine samples	miR-30b-5p	MAPK signaling pathway	low	N/A	Guo et al. (2022)
	miR-9-5p				
serum samples	chrY:13688616|13833086	platelet activation signaling pathway	high	N/A	Ma et al. (2019)
	chr2:233244474|233272478				
urine samples	chrY:13842647|13855594	P13K/Akt signaling pathway	high		
	chrY:13688616|13833086		low		

MAPK, mitogen-activated protein kinase; miR, microRNA; N/A, not available; ncRNA, non-coding RNA; PI3K, phosphoinositide-3-kinase-protein kinase B; sEVs, small extracellular vesicles.

### 5.1 Roles of sEV-miRNAs in IMN

Presently, only a few scholars are exploring the diagnostic value of sEV-miRNAs in IMN. Guo et al. (2022) investigated the expression profile of urinary sEV-miRNA in IMN patients and found that miR-30b-5p and miR-9-5p were markedly downregulated in patients with IMN as compared to healthy controls. In their study, the potential diagnostic value for IMN of miR-30b-5p and miR-9-5p was also demonstrated by the authors through ROC curve analysis.

### 5.2 Roles of sEV-circRNAs in IMN

Only a few studies exist assessing the diagnostic potential of sEV-circRNAs on IMN. In the study by Ma et al. (2019), the comparison of serum sEV-circRNAs between IMN patients and healthy controls found 89 differentially expressed sEV-circRNAs, 49 of which were upregulated and 40 of which were downregulated. In detail, chrY:13688616|13833086 was the most prominent upregulated circRNA, while chr2:233244474|233272478 was the most prominent downregulated circRNA. In addition to serum sEV-circRNAs, the authors found 60 urinary sEV-circRNAs with significantly differentially expression, of which 54 expressions were upregulated and 6 expressions were downregulated. Among these, chrY:13842647|13855594 was the most prominent upregulated circRNA, while chrY:13688616|13833086 was the most prominent downregulated circRNA. As discussed above, sEV-circRNAs may be useful diagnostic biomarkers for kidney disease.

## 6 Roles of sEV-miRNAs in Renal fibrosis

Renal fibrosis is an unavoidable progressive component of virtually all forms of chronic kidney diseases, culminating in renal failure. sEV-miRNAs were considered to correlate with renal fibrosis (Abbasian et al., 2018) (Table 5).

**TABLE 5:** The role of small extracellular vesicle non-coding RNAs in renal fibrosis.

Origin of sEVs	sEVs cargo	Pathway	ncRNA expression	Mechanism	References
urine samples	miR-29c	N/A	low	have good accuracy in the diagnosis of renal fibrosis	Chun-Yan et al. (2018)
urine samples	miR-29c	N/A	low	have good accuracy in the diagnosis of renal fibrosis	Lv et al. (2013)
MSCs	miR-let7c	TGF-βR1	high	attenuate renal fibrosis	Wang et al. (2016)
serum samples	miR-26a	CTGF	high	attenuate UUO-induced renal fibrosis	Zhang et al. (2019)

CTGF, connective tissue growth factor; miR, microRNA; MSCs, mesenchymal stem cells; N/A, not available; ncRNA, non-coding RNA; sEVs, small extracellular vesicles; TGF-βR1, transforming growth factor-β type 1 receptor; UUO, unilateral ureteral obstruction.

Renal fibrosis is primarily caused by EMT. miR-29c was found to participate in the process mentioned above, and therefore, it was proposed to support an early diagnosis of renal fibrosis. For instance, Lv et al. (2013) found that urine sEV-derived miR-29a and miR-29c levels were significantly lower in moderate-to-severe renal fibrosis patients than mild ones. Both the aforementioned miRNAs were of predictive value in the assessment of degree of renal fibrosis, with an AUC of 0.883 and 0.738. In addition, further analysis revealed that miR-29c is significantly correlated with fibrosis severity. Similar to these earlier findings, another study demonstrated that a considerable decrease in urine miR-29c was measured in patients with renal fibrosis when compared with controls (Chun-Yan et al., 2018). Moreover, AUC of miR-29c in diagnosis of renal fibrosis was 0.862. These findings gave credence to the notion that miR-29c had the potential to be introduced as a new diagnostic marker in the future.

The significant role that sEVs play in kidney diseases has prompted researchers to explore them as promising therapeutics. The mouse unilateral ureteral obstruction (UUO) model has been extensively utilized in studies of renal fibrosis, since it offers obvious advantages of rapid renal fibrosis development by induction (Wu et al., 2017). Zhang et al. (2019) demonstrated in the mouse UUO model that serum miR-26a combated renal fibrosis by inhibiting connective tissue growth factor (CTGF). Moreover, Wang et al. (2016) observed that miR-let7c was transported to damaged kidney cells *via* sEVs from MSCs, thus slowing renal fibrosis progression.

## 7 Discussion

CKD is one of the most frequent chronic disease worldwide, which is characterized by its high prevalence and low awareness (Zhang et al., 2012; Al Rahbi and Al Salmi, 2020). Once CKD progresses to ESRD, patients tend to have a dismal prognosis. As such, developing an *in vitro* testing technique at the same time possessing high sensitivity and high specificity are fundamentally important to achieving early detection, intervention, as well as good prognosis for the patients with kidney diseases. A convincing number of studies have confirmed that sEV-ncRNAs participate in the regulation of numerous cellular functions and play an essential role in the development of kidney disorders during the recent years. Also, using serum and urine samples to diagnose kidney diseases has the advantage of easy access, as well as being non-invasive, and reproducible, which are beneficial for achieving ambulatory monitoring of disease progression. As a result, liquid biopsies based on sEVs have begun to show its great benefits and application prospects in diagnosing kidney disorders.

The present review provide a thorough summary of the role of sEV-ncRNAs (including miRNAs, lncRNAs and circRNAs) in kidney disorders. Currently, the study addressing the relationship between sEV-ncRNAs and kidney disorders focuses mainly on the following four aspects. Firstly, a significant difference exists between healthy people and patients with kidney disease regarding the composition and contents of sEV-ncRNAs (Bai et al., 2020; Ling et al., 2019). Thus, we speculate that sEV-ncRNAs contribute to the initiation and development of kidney diseases, although the precise mechanism remains to be determined. Secondly, sEV-ncRNAs have shown an exceptional value for diagnosing kidney diseases (Li et al., 2022; Guo et al., 2022). At present, the global research efforts primarily focus on exploring the possibility of using sEV-ncRNAs as diagnostic tools for kidney diseases. However, it still requires considerably more work to make sEV-ncRNA a rountine diagnostic method in clinical practice. Thirdly, the therapeutic potential of sEV-ncRNAs in treating kidney diseases is relatively understudied (Jin et al., 2019). More work is required to determine how can they be used in the field of kidney disease therapy in the future. Fourthly, although there is a growing number of studies demonstrating the potential for sEV-ncRNAs to serve as prognostic biomarkers in patients with kidney disorders, the total amount of relevant research remains limited (Fan et al., 2019).

Despite tremendous advances in our understanding of the role of sEV-ncRNAs in kidney diseases in recent decades, the research in this regard is still in its nascent stage with many areas that can be improved upon. Firstly, since sEV-ncRNAs have been studied primarily in the above commonly prevalent kidney diseases, there is a need to examine their role in other kidney diseases before a consensus about their role in the progression of kidney diseases can be reached. Secondly, there was at least one methodological flaw in most of the studies reviewed, primarily that their sample sizes were too small, which may influence the reliability of the outcomes. Thus, it will be necessary to confirm these preliminary results with large-scale and well-designed studies. Thirdly, there has been an increase in research exploring how sEV-miRNAs contribute to kidney diseases over the past few years. Contrary to this, studies examining sEV-lncRNAs or sEV-circRNAs in kidney disorders are relatively rare, which offers a possible entry point for future research. Last but not least, in spite of promising early evidence, sEV-ncRNAs still suffer from several major difficulties in terms of clinical application. On the one hand, owing to the limited quantities of genetic material in the sEVs, how to make the best use of sEV-ncRNAs become an urgent problem that needs to be solved. On the other hand, it is still unclear how molecules are synthesized and packaged into sEVs, so further, more elaborate research is needed.

## 8 Conclusion

In summary, sEV-ncRNAs play fundamental roles in the early detection, treatment and prognosis of kidney disease. With the gradual maturity of experimental techniques, sEV-ncRNAs have become one of the most potential candidate diagnostic and prognostic biomarkers and therapeutic targets of kidney diseases. This review merely scratches the tip of the iceberg referring to sEV-ncRNAs in kidney diseases. Several have yet to be explored, while others deserve further investigation. Intensive investigation of sEV-ncRNAs could lend insights into the biological functions and regulatory mechanisms of sEVs, and hopefully reveal new interactive molecules and signaling pathways, providing new insight into the diagnostic and therapeutic approaches to kidney disease.

## Glossary

ADSCs: adipose-derived stem cells
AKI: acute kidney injury
AP-1: activator protein 1
ASCL4: acyl-CoA synthetase long-chain family member 4
AUC: area under the curve
circRNAs: circular RNAs
CKD: chronic kidney disease
CTGF: connective tissue growth factor
DKD: diabetic kidney disease
EMT: epithelial interstitial transition
EPCs: endothelial progenitor cells
ERBB3: Erb-b2 receptor tyrosine kinase 3
ESRD: end-stage renal disease
GECs: glomerular endothelial cells
GMCs: glomerular mesangial cells
GO: gene ontology
hPMSCs: human placental MSCs
IgAN: IgA nephropathy
IGF1R: insulin-like growth factor 1 receptor
IMN: idiopathic membranous nephropathy
IRAK1: interleukin-1 receptor-associated kinase 1
IRI: ischemic/reperfusion injury
KEGG: Kyoto Encyclopedia of Genes and Genomes
lncRNAs: long non-coding RNAs
LPS: lipopolysaccharide
MAPK: mitogen-activated protein kinase
miRNAs: microRNAs
MMP2: matrix metalloproteinase 2
MSCs: mesenchymal stem cells
mTOR: mechanistic target of rapamycin
ncRNAs: non-coding RNAs
NF-κB: nuclear factor-κB
OXSR1: oxidative stress responsive 1
PI3K: phosphoinositide-3-kinase-protein kinase B
PRB: percutaneous renal biopsy
ROC: receiver operating characteristic
RUNX1: runt-related transcription factor 1
sEV-circRNAs: small extracellular vesicle-derived circular RNAs
sEV-lncRNAs: small extracellular vesicle-derived long non-coding RNAs
sEV-miRNAs: small extracellular vesicle-derived microRNAs
sEV-ncRNAs: small extracellular vesicle-derived non-coding RNAs
sEVs: small extracellular vesicles
Smad1: SMAD family member 1
Smad3: SMAD family member 3
SOCS-1: suppressor of cytokine signaling 1
SRSF1: serine/arginine splicing factor 1
T1DM: type 1 diabetic mellitus
T2DM: type 2 diabetes mellitus
TECs: tubular epithelial cells
TGF-β: transforming growth factor-β
USCs: urine-derived stem cells
UUOunilateral ureteral obstructionZEB2: unilateral ureteral obstructionZEB2zinc finger E-box-binding homeobox 2
